# The influence of muscle, ageing and thermal treatment method on the quality of cooked beef

**DOI:** 10.1007/s13197-021-04993-x

**Published:** 2021-02-08

**Authors:** Monika Modzelewska-Kapituła, Katarzyna Tkacz, Zenon Nogalski

**Affiliations:** 1 Department of Meat Technology and Chemistry, Faculty of Food Sciences, University of Warmia and Mazury in Olsztyn, Plac Cieszyński 1, 10-719, Olsztyn, Poland; 2 Department of Cattle Breeding and Milk Evaluation, Faculty of Animal Bioengineering, University of Warmia and Mazury in Olsztyn, Oczapowskiego 5, 10-719, Olsztyn, Poland

**Keywords:** Cattle, Colour, Cooking loss, Meat, Tenderness, Sensory quality

## Abstract

The study was undertaken to investigate the effect of muscle, thermal treatment, and ageing on the beef quality. The *longissimus lumborum* (LL) and *semimembranosus* (SM) muscles were taken from Holstein–Friesian young bull carcasses then subjected to steam-cooking and sous-vide after 9 and 14-d wet ageing. It was shown that characteristics of cooked beef were the most significantly affected by thermal treatment method. Using sous-vide provided beef with lower shear force and cooking loss values, darker, and more red colour and more beneficial sensory quality. LL and SM muscles showed a similar quality when subjected to the same thermal treatment method after the same ageing time. It is possible to obtain juicy and tender beef from Holstein–Friesian bulls after 9-d ageing and sous-vide treatment.

## Introduction

Among the European Union countries, Poland ranks seventh in terms of cattle population. In December 2018, the cattle population was 2.4% higher than in the previous year and was estimated to be 6,183,000 head. The gradual increase in the cattle population as well as the increase in citizens' income may increase beef consumption. However, the level of beef consumption in Poland is still lower than the average level in the European Union. In the European Union, the average inhabitant consumes 10–11 kg of beef a year, while in Poland it is estimated to be 3.3 kg (Anonymous 2019). The low level of beef consumption is due, in part to the high price of meat, but also due to consumers' concerns about beef quality (Żakowska-Biemans et al. 2017). There is a general opinion that beef, especially from dairy or dual-purpose cattle, which is predominant in Poland, is not tender nor juicy. The treatment that can overcome the problem of insufficient tenderness of beef is post-slaughter ageing (Colle et al. 2015). This treatment can be applied to large cuts, such as half carcasses, or smaller parts, such as individual steaks.

The ageing method, which can be easily applied to muscles or muscle parts, is wet ageing, during which beef packed in a vacuum is stored at refrigerated temperature. Vacuum-packing of muscles, muscle cuts, or individual steaks can be done by meat producers, retailers, but also in restaurants or households due to the high availability of devices for vacuum-packaging and their relatively low price. The ageing time may vary, depending on the muscle type or the place the beef is stored. Guelker et al. (2013) reported that an average ageing (storage) time at foodservice establishments was 28 days, and ranged from 9 to 67 days. Colle and Doumit (2017) reported that consumer perception of the tenderness of *longissimus lumborum* (LL) did not increase when the muscle was aged longer than 14 days, whereas in the case of *semimembranosus* (SM) it was higher after 42 days of aging than after 14 days.

Another approach for obtaining tender and juicy ready-to-eat beef is applying an appropriate thermal treatment method (Guzek et al. 2015). Sous-vide is a relatively novel thermal treatment where a juicy and tender meat may be obtained from almost all meat cuts (Baldwin 2012). It is gaining popularity among chefs of restaurants and is used on the industrial scale for producing convenient food, which is ready for consumption after short heating by a consumer. In this technique, meat is vacuum-packed and subjected to heating in a water bath at a temperature from 50 to 70 °C for several hours (Dominguez-Hernandez et al. 2018). This technique has many advantages, such as obtaining good tenderness of meat (Baldwin 2012), inhibition of lipids oxidation (Vaudagna et al. 2002), the possibility to store meat after sous-vide treatment for a long time at refrigerated temperature, reduction of colour changes and inhibition of heterocyclic aromatic amines formation in meat products (Oz and Zikirov 2015). However, there are also some concerns related to safety, such as the possibility that a thermally-resistant bacterium e.g. *Clostridium botulinum* spores (Vaudagna et al. 2002) survives, and the migration of various components of plastic bags in which products are heated, including bisphenol-A (BPA), into the food during heat treatment (Oz and Seyyar 2016). Another thermal treatment method, invented to obtain juicy and tender meat products, is steam-cooking. The thermal treatment proceeds in the environment of steam, which is injected into the oven chamber. The use of steam reduces the time of thermal treatment and protects the meat products from surface dehydration as compared to heating in dry air (Isleroglu et al. 2015; Modzelewska-Kapituła et al. 2012).

Beef carcass muscles differ in their chemical composition, physicochemical properties, and eating quality. *Longissimus lumborum *(LL) muscle is widely used for culinary purposes and has desirable sensory quality, whereas *semimembranosus* (SM) muscle has a uniform appearance, which can attract consumers, but is less tender than LL muscle (Colle et al. 2018). The differences between the muscles result from their localization in the beef carcass and the functions in a live animal, which in turn produced differences in the composition, including muscle fibre characteristics, fat, and collagen content. According to the authors' best knowledge, there are no reports showing the influence of aging and wet thermal treatment methods, such as steam cooking and sous-vide, on the quality of LL and SM muscles from dairy cattle. Therefore, the aim of the present study was to investigate the effect of muscle type, ageing, and thermal treatment method on tenderness and other beef quality attributes. Moreover, the hypothesis that the tenderness of SM muscle might be similar to LL muscle when an appropriate combination of aging time and thermal treatment method is used, was tested.

## Material and methods

### Material

The study was conducted using *longissimus lumborum* (LL) and *semimembranosus* (SM) muscles originating from the carcasses of young Holstein–Friesian black and white variety bulls, (n = 12, the average age at slaughter 20.0 ± 0.6 months). The protocol for animal studies was approved by the Ethics Committee of the University of Warmia and Mazury in Olsztyn (decision no. 121/2010). The bulls were fattened semi-intensively, receiving ad libitum a total mixed ration composed of maize silage with the addition of a concentrate containing rapeseed and triticale meals and premix of vitamins and minerals. The fattening was finished when the animals reached 600 kg of body weight. After delivering to an abattoir the bulls rested for 15 to 20 h in individual boxes with free access to water. The bulls were slaughtered in compliance with Council Regulation (2009) (EC) No 1099/2009 of 24 September 2009 on the protection of animals at the time of killing. Electrical stimulation was not used. After slaughter, the beef carcasses were cooled using a one-stage method (air temperature 1°C, flow rate 0.5 m/s, 90% RH) for 24 h to obtain 4°C in the centre of the hindquarter muscles. Before and after slaughter the animals were weighed with an accuracy of 0.5 kg.

### Muscle sampling

Muscles LL (n = 12) and SM (n = 12) were removed from left half-carcasses of each animal 24 h post mortem and then transported (about 3 h) to a laboratory in a portable fridge cooler at refrigerated temperature. After delivery to the University’s laboratory, the muscles were placed into a refrigerator (4 ± 1 °C) and kept overnight. Then, each muscle (mean weight 1230.8 g ± 20.9 g) was split into two cuts (one for 9-day and another for 14-day ageing), which were packed individually under vacuum in PA/PE bags (Inter Arma sp. z o.o., Rudawa, Poland; thickness 70 µm, total transmission rates not exceeding 10 mg/dm^2^ for model liquids, 3% acetic acid, 50% ethyl alcohol for 10 days at 40 °C and isooctane for 2 days, 20 °C). Beef samples were stored until the 9th and 14th-day post mortem at 4 ± 1 °C in a climate chamber (Memmert GmbH, Schwabach, Germany). Then, the meat was removed from the vacuum, and cut into 2.5 cm thick steaks. From each sample, one steak was steam-cooked and the other was subjected to sous-vide treatment. In cooked samples cooking loss, Warner–Bratzler Shear Force (WBSF), colour and sensory quality attributes were determined. The proximate chemical composition of raw beef was determined in the samples (approx. 300 g) aged until the 14th-day post mortem.

### Proximate chemical composition, and pH of raw meat

After removing the external fat and thick connective tissue from the muscles, the meat was ground twice using a 3 mm mesh and then thoroughly manually mixed. Values of pH were measured with a combined electrode FC 200 and pH-meter HI 8314 (Hanna Instruments Polska, Olsztyn, Poland). The device was first calibrated using pH 7 and pH 4 buffers. Moisture was determined according to PN-ISO^1442^ (2000) (drying at 103 ± 2 °C to constant weight), fat according to AOAC no 991.36 (2006a), protein according to AOAC no 992.15 (2006b), and ash according to PN-ISO 936 (2000). The analyses were done in triplicate for each sample.

### Thermal treatment

Each muscle from each carcass was subjected to both thermal treatment methods: steam-cooking and sous-vide. Steam-cooking was performed in a preheated convectional-steam oven (Retigo Vision 623i, Retigo, Rožnov pod Radhoštěm, Czech Republic), by heating the steaks in 100% steam environment at 100 °C to obtain an internal temperature of 75 °C. A thermometer connected to the oven was used to monitor the internal temperature of the steaks. The steaks intended for sous-vide treatment were packed individually in PA/PE bags (Inter Arma sp. z o.o., Rudawa, Poland) suitable for heating food products and cooked in a sous-vide device (Fusion Chef by Julabo Diamond Z, Julabo GmbH, Seelbach, Germany) at 60 °C for 4 h. To determine cooking loss, steam cooked and sous vide-steaks were weighed before and after cooking.

### Colour

The colour of the beef was measured before and after thermal treatment on the surface of steaks in the CIE L*a*b* system, with a MiniScan XE Plus device (HunterLab, Reston, USA) with standard illuminant D65, a 10° standard observer angle and a 2.54-cm-diameter aperture. Raw steaks before thermal treatment were left on trays in a refrigerator at 4 ± 1 °C for 1 h to allow blooming and then the colour was measured. The colour of cooked steaks was measured 10 min. after the thermal treatments had finished, after the meat surface had been dried with a paper towel. Prior to the measurement readings, the device was calibrated using white and black tiles supplied by the manufacturer. Colour was measured in three different locations of the surface of each steak. To better highlight differences in colour, the indexes of hue angle (H) of the samples H = arctangent (b*/a*)·360°/(2·Π) and chroma (C) C = (a*^2^ + b*^2^)^0.5^ were calculated. To determine the colour difference between raw and cooked steaks, ∆E coefficient, indicating the total colour change between raw and cooked beef, was calculated using the formula: ∆E = [(∆CIE L*)^2^ + (∆CIE a*)^2^ + (∆CIE b*)^2^]^0,5^ (CIE 1978), where ΔCIE L*, ΔCIE a* and ΔCIE b* are the difference in values obtained after and before thermal treatment for individual steaks.

### Warner–Bratzler shear force values (WBSF)

The samples for WBSF determination were cut from steam-cooked and sous-vide meat parallel to the longitudinal orientation of the muscle fibres after overnight chilling (3 ± 1 °C). The samples (10 mm × 10 mm, about 40 mm long, n = 5 from each steak) at room temperature (approx. 20 °C) were cut perpendicular to the longitudinal orientation of the muscle with a shear blade with a triangular aperture of 60° (load 500 N, head speed 200 mm/min, Instron 5942, Instron, Norwood, USA).

### Sensory assessment

The sensory assessment was performed on the LL and SM samples aged for 14 days. As soon as the thermal treatments were finished, samples were cut into approx. 2-mm thick slices, coded with three-digit numbers, and served to the panellists (n = 6, trained for 36 h, non-smokers, females) randomly on white plates. Panellists scored each sample for colour uniformity (1, very uneven, 10, entirely even), aroma intensity (1, imperceptible; 10, extremely intense) and its acceptability (1, not acceptable; 10, very desirable), juiciness (1, extremely dry; 10, extremely juicy), tenderness (1, extremely tough; 10, extremely tender), meat taste intensity (1, imperceptible; 10, extremely intense) and its acceptability (1, not acceptable; 10, very desirable and overall acceptability (1, not acceptable; 10, very desirable) using a structured scale. In total, 4 sensory analysis sessions were performed during which a maximum of 6 meat samples was evaluated. The sessions were carried out at room temperature (approx. 20 °C) under fluorescent lighting. Water and bread were used to cleanse the palate. At the same time, 3 samples were presented followed by an approx. 20 min interval before the assessment of the next samples.

### Data analysis

Statistical analysis of the gathered data was performed using Statistica 13.3 (TIBCO Software Inc., Palo Alto, CA., USA) software. The results were presented as mean values and standard error of the mean. To examine the differences between mean values obtained, excluding sensory analysis results, an analysis of variance was conducted, and Duncan’s test. To compare sensory analysis results, non-parametric U Mann–Whitney and Kruskal–Wallis tests were applied to compare two and more groups of means, respectively. The significance level was set at 0.05. Mixed model ANOVA/ANCOVA (variation components module, with df error calculated with Satterthwaite’s method) was used to determine the effect of muscle (two levels: LL and SM), ageing (two levels: 9 and 14 days) and thermal treatment method (two levels: sous-vide and steam cooking). Muscle type, ageing and thermal treatment method were identified as fixed factors, and carcass was categorized as a random effect. Cluster analysis was used to classify objects into groups using data from WBSF determination, cooked meat colour (L*, a*, b*, C and H values), and cooking loss.

## Results and discussion

### Raw beef characteristics

LL muscles contained 73.9% moisture, 21.65% protein, 2.95% fat, 1.14% ash, and SM muscles 75.18%, 22.05%, 1.02% and 1.22%, respectively. Chemical composition of raw beef was similar to that noted by Wyrwisz et al. (2016) and Bureš and Bartoň (2018) for meat obtained from dairy cattle (Holstein–Friesian or Holstein). LL and SM muscles differed (*P* < 0.05) in their chemical composition (excluding ash) and pH, which was expected due to different locations in beef carcass and functions in a live animal. The pH values of both muscles were 5.70 and 5.72 for LL and SM, respectively, and were typical for normal quality beef.

### Characteristics of cooked beef–instrumental measurements

#### WBSF

In both muscles lower (*P* < 0.05) WBSF values were noted in sous-vide samples compared with steam-cooked (Table 1). The samples of SM muscle aged for 14 days showed significantly (*P* < 0.05) lower values than 9-d aged, regardless of the thermal treatment method (Table 1). This indicates that longer ageing increased the tenderness of SM muscles subjected to sous-vide and steam-cooking. However, in the case of LL, this trend was not noted. There were no differences noted in WBSF between LL and SM muscles subjected to the same thermal treatment method at the same ageing time. The effects of ageing (*P* < 0.05) and thermal treatment method (*P* < 0.001) on WBSF values are shown in Table 2. Generally sous-vide beef had lower WBSF values than steam-cooked beef and 14-d aged beef was lower than 9-d aged. Sous-vide cooking is generally regarded as a thermal treatment which provides tender meat, so the effect was expected.

**Table 1:** Warner–Bratzler Shear Force (WBSF), cooking loss and colour of *longissimus lumborum* (LL) and *semimembranosus* (SM) muscles aged for 9 and 14 days after thermal treatment

Attribute	Muscle	Ageing/thermal treatment			
		9 days		14 days	
		Sous-vide	Steam-cooking	Sous-vide	Steam-cooking
WBSF (N)	LL	38.6 (2.3)^bx^	51.0 (2.9)^ax^	36.5 (1.7)^bx^	47.8 (2.6)^ax^
	SM	40.5 (1.3)^cx^	52.2 (2.5)^ax^	35.0 (1.1)^dx^	47.3 (1.6)^bx^
Cooking loss (%)	LL	17.8 (0.5)^cy^	26.9 (0.8)^ay^	20.9 (0.8)^by^	29.1 (0.7)^ay^
	SM	26.8 (0.7)^bx^	36.7 (0.8)^ax^	27.5 (0.9)^bx^	33.9 (0.9)^ax^
*Colour*					
L*	LL	36.2 (0.6)^cx^	50.1 (0.6)^ax^	38.2 (0.7)^by^	51.1 (0.6)^ax^
	SM	34.1 (0.7)^cy^	49.2 (0.5)^ay^	44.0 (0.9)^bx^	49.2 (0.3)^ay^
a*	LL	8.58 (0.23)^ay^	4.51 (0.07)^cx^	8.00 (0.25)^by^	4.36 (0.08)^cx^
	SM	10.1 (0.4)^ax^	4.04 (0.15)^cy^	9.11 (0.24)^bx^	4.12 (0.11)^cx^
b*	LL	15.83 (0.28)^abx^	15.53 (0.17)^bx^	15.23 (0.26)^by^	16.25 (0.16)^ax^
	SM	16.7 (0.7)^abx^	15.67 (0.16)^cx^	17.4 (0.3)^ax^	15.97 (0.15)^bcx^
C	LL	18.0 (0.3)^ay^	16.18 (0.16)^cx^	17.2 (0.3)^by^	16.83 (0.15)^bcx^
	SM	19.6 (0.8)^ax^	16.19 (0.15)^bx^	19.7 (0.3)^ax^	16.50 (0.16)^bx^
H	LL	61.6 (0.6)^bx^	73.74 (0.29)^ay^	62.4 (0.6)^bx^	74.9 (0.3)^ax^
	SM	58.9 (0.4)^cy^	75.5 (0.5)^ax^	62.9 (0.7)^bx^	75.6 (0.4)^ax^
∆E	LL	11.2 (0.4)^dy^	19.8 (0.4)^by^	12.9 (0.3)^cy^	21.7 (0.5)^ax^
	SM	12.9 (0.7)^cx^	23.4 (1.1)^ax^	15.8 (0.5)^bx^	21.8 (0.4)^ax^

^a^^−^^d^mean values in rows with different letters differ significantly at *P* < 0.05; ^x,y^mean values in columns within each attribute with different letters differ significantly at *P* < 0.05; ∆E difference in colour before and after thermal treatment

**Table 2:** Influence of muscle, ageing and thermal treatment method on the quality of beef steaks (mean values and standard error of the mean in parentheses)

Attribute	Muscle (M)		Ageing (A)		Thermal treatment (T)		*P* level						
	LL	SM	9	14	Sous-vide	Steam-cooking	M	A	T	MxA	MxT	AxT	MxAxT
WBSF (N)	41.9 (1.2)	42.7 (0.9)	44.6 (1.3)	40.9 (0.9)	37.2 (0.8)	49.1 (1.2)	NS	*	***	NS	NS	NS	NS
Cooking loss (%)	24.5 (0.7)	31.7 (0.8)	25.9 (1.0)	28.8 (0.7)	22.7 (0.7)	31.0 (0.6)	***	*	***	*	NS	NS	NS
*Colour*													
L*	43.9 (0.6)	44.9 (0.6)	42.6 (0.8)	45.6 (0.5)	38.7 (0.5)	49.98 (0.26)	NS	***	***	NS	***	*	***
a*	6.37 (0.18)	6.77 (0.28)	6.72 (0.26)	6.4 (0.2)	8.79 (0.14)	4.29 (0.05)	NS	NS	***	NS	***	NS	NS
b*	15.71 (0.11)	16.52 (0.17)	15.85 (0.16)	16.21 (0.13)	16.23 (0.19)	15.88 (0.09)	*	NS	*	NS	*	NS	NS
C	17.07 (0.13)	18.02 (0.24)	17.36 (0.22)	17.56 (0.16)	18.49 (0.21)	16.46 (0.08)	*	NS	***	NS	***	NS	NS
H	68.2 (0.6)	68.4 (0.8)	67.5 (0.7)	68.8 (0.6)	38.7 (0.5)	49.98 (0.26)	NS	*	***	NS	NS	NS	*
∆E	16.4 (0.4)	18.6 (0.5)	16.4 (0.5)	18.1 (0.4)	13.26 (0.28)	21.45 (0.27)	***	*	***	NS	NS	*	**

LL *longissimus lumborum*; SM *semimembranosus*; *** *P* < 0.001; ** *P* < 0.01; * *P* < 0.05; NS no significant difference, ∆E difference in colour before and after thermal treatment

Based on WBSF values, beef might be classified on different grades of tenderness: very tender (WBSF below 32.96 N), tender (WBSF from 32.96 N to 42.77 N), acceptably tender (WBSF from 42.87 N to 52.68 N), hard (WBSF from 52.78 N to 62.59 N) and very hard (WBSF above 62.59 N) (Destefanis et al. 2008). Results obtained in the present study indicate that 9 and14-d aged sous-vide LL and SM were tender, whereas all steam-cooked samples, regardless of the ageing time were acceptably tender. The differences between sous-vide and steam-cooked samples resulted from the level of temperature obtained in steaks and the duration of thermal treatment. Sous-vide treatment was processed at a lower temperature (60 °C) and a much longer time (4 h) than steam-cooking (75 °C and approx. 25 min., respectively). Along with a thermal treatment time the collagen solubility and gelatinization increase (Purslow 2014), which in turn results in weakening the structure of connective tissue and reduced WBSF values (Baldwin 2012). Moreover, with the increase in internal meat temperature during cooking, meat became less tender. In the range from 65 °C to 75 °C the most significant and rapid increase in WBSF is noted (Li et al. 2010) as a result of myofibrillar protein denaturation (Dominguez-Hernandez et al. 2018), which was another reason for obtaining higher WBSF values for steam-cooked samples in this study.

#### Cooking loss

In LL muscle, the lowest cooking loss was noted after sous-vide treatment of 9-d aged meat, however, all sous-vide samples showed lower cooking loss than steam-cooked (*P* < 0.05), which was also noted in SM muscle (Table 1). Moreover, for every ageing time and both thermal treatments, LL exhibited a lower cooking loss than SM. The multifactorial analysis showed a significant effect of muscle, ageing, and thermal treatment method on cooking loss and interaction between ageing and muscle (Table 2). The differences in the cooking loss between sous-vide and steam-cooked beef resulted from the differences in the internal temperature to which meat was heated. With the increase in temperature cooking loss increased, and thus steam-cooked samples showed a higher cooking loss than sous-vide beef. The relation between the internal temperature of meat and cooking loss was also reported by Alfaia et al. (2010) and Purslow et al. (2016). Additionally, vacuum packing of beef in plastic bags prior to sous-vide treatment prevented moisture evaporation and thus decreased cooking loss (Baldwin 2012). Lower cooking loss of LL compared with SM might be explained by differences in the chemical composition, such as lower moisture and higher fat content, and the fact that meat with a higher fat content shows a lower cooking loss (Lawrence et al. 2001; Modzelewska-Kapituła et al. 2012). In the present study, increasing the aging time from 9 to 14 days increased the cooking loss (Table 2). A similar trend was shown by Purslow et al. (2016) in beef samples aged for 1 and 14 days. The changes in cooking losses as a result of ageing might be attributed to the reduced stability of myofilaments, which in turn is demonstrated by the greater denaturation at particular cooking temperature and higher shrinkage of muscle samples which determines cooking losses (Purslow et al. 2016).

#### Colour

Sous-vide LL and SM steaks were darker (lower L* values) than steam-cooked samples in every sampling time. Moreover, LL steaks had higher lightness (L*) than SM, except for the samples subjected to sous-vide after 14-d ageing, where opposite was noted (Table 1). The lowest values of L* were noted in both muscles after 9-d ageing and sous-vide cooking. Sous-vide steaks showed higher redness (a* values) and lower hue angle in LL and SM muscles. However, the b* and C colour parameter values varied between samples at different ageing times and thermal treatment (Table 1). Similar results were reported by García-Segovia et al. (2007) who noted higher a* and b* values in sous-vide beef steaks compared to atmospheric pressure cooked samples. Generally, meat type affected yellowness (b*), and chroma (C), ageing affected lightness (L*) and hue angle (H) values, whereas thermal treatment method affected all of the colour parameters (Table 2).

The results obtained clearly showed that the thermal treatment method affects the colour of cooked beef more than muscle type and ageing time. The lighter surface colour of steam-cooked beef might result from greater changes in meat proteins, due to a higher ambient temperature during the thermal treatment (100 °C and 60 °C for steam-cooking and sous-vide, respectively), such as protein precipitation and loss of their solubility (Ledward 1992, as cited by Sun et al. 2019). Another reason for observed differences between sous-vide and steam-cooked beef was the degree of myoglobin denaturation which starts between 55 and 65 °C, and that most of the myoglobin is denatured and brown at 70 °C to 80 °C (Hunt et al. 1999). Myoglobin denaturation occurred more easily under the atmospheric cook conditions (García-Segovia et al. 2007), as oxymyoglobin is less stable than deoxymyoglobin (Sun et al. 2019). Higher values of a* indicating lower metmyoglobin content in sous-vide samples resulted from vacuum packaging of the samples prior to cooking and reduced oxygen content, which in turn limited the myoglobin oxidation and formation of metmyoglobin. The differences in yellowness might result from enzymatic deactivation and protein denaturation (Sun et al. 2019) and therefore samples of sous-vide cooked at lower temperature showed higher b* values. The consequences of the differences in a* and b* values were higher chroma and lower hue angle of sous-vide beef, which indicates higher colour saturation and more red and less yellow appearance than steam-cooked samples.

Due to the fact that cooking results in changes in the appearance of meat, in the present study ΔE coefficient, indicating the total colour change between raw and cooked beef, was calculated. Generally, values higher than 2 indicate that the colour change will be noticed even by an unexperienced observer (CIE 1978)*.* The higher the ΔE value, the greater and more numerous the changes in beef colour between the raw and cooked meat will be perceived by consumers. In all treatments, ΔE values were higher than 2 (Table1), which was expected and indicated that consumers would easily distinguish between raw and sous-vide or raw and steam-cooked beef samples. However, in both muscles higher values were noted for steam-cooked beef and longer ageing time. SM showed higher values than LL (except for 14-d aged steam-cooked steaks). All the factors (muscle, ageing, and thermal treatment method) affected the ΔE values and interaction between ageing and treatment, and between all three factors were noted (Table 2). Generally, lower changes in colour between raw and cooked beef steaks were noted for LL muscle, 9-d ageing, and sous-vide treatment. This might result from muscle chemical composition, muscle composition (muscle fibres, connective tissues, adipose tissues, vascular, and nervous tissues), and changes in myoglobin forms during ageing and meat protein denaturation during thermal treatment.

**Fig. 1:**
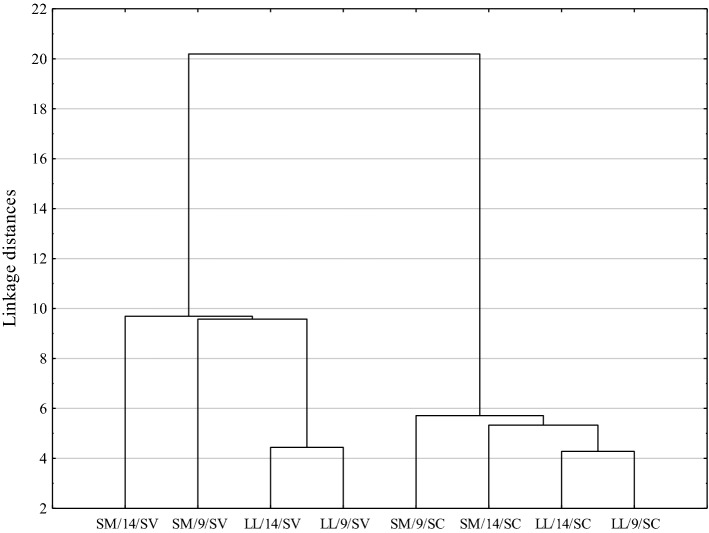
Complete-linkage dendrogram for steam cooked (SC) and sous-vide cooked (SV) *longissimus lumborum* (LL) and *semimembranosus* (SM) muscles aged for 9 and 14 days

A dendrogram of cooked beef samples, using a Euclidean distance as a measure of the proximity between samples is shown in Fig. 1. The dendrogram based on WBSF, cooking loss, and colour of experimental data showed the distinction between sous-vide and steam-cooked beef samples. The variability among samples is due to the method of thermal treatment and muscle type rather than ageing, which supports the findings that the method of thermal treatment determines the quality of beef more than ageing and muscle type.

####  Sensory quality

Results of a sensory assessment of LL and SM steaks subjected to sous-vide and steam-cooking after 14-d ageing are shown in Table 3. There were no differences noted between treatments and muscles for colour uniformity on the steaks’ surface. Steam-cooked LL muscles showed higher scores for meat aroma intensity and acceptability, however, no differences were noted in SM. Sous-vide LL and SM steaks were juicier and more tender then their steam-cooked counterparts and no differences were noted between LL and SM. This supports the results of instrumental analysis, where the lack of the differences in WBSF values between sous-vide LL and SM steaks for 14-d aged beef and lower WBSF values for sous-vide beef were noted. The differences in taste acceptability and intensity were noted only for SM muscles, and sous-vide samples were scored higher. Sous-vide samples were also scored higher in terms of overall acceptability than steam-cooked samples. Moreover, sous-vide steaks from SM were scored higher than LL (Table 3). Generally, muscle type affected meat aroma intensity and acceptability and juiciness, whereas the method of thermal treatment affected meat aroma intensity and acceptability, juiciness, tenderness, meat taste intensity, acceptability, and overall acceptability (Table 4). LL muscle was scored higher than SM for aroma and juiciness, which resulted from higher fat content in raw beef. Sous-vide beef was scored higher for juiciness and tenderness than steam-cooked samples but lower for meat aroma intensity and acceptability. The results might be explained by the fact that meat aroma, which results from the volatile aromatic compounds, develops at temperatures above 70 °C (Calkins and Hodgen 2007). This was noted also by Park et al. (2020), who reported lower scores for flavour and off-flavours in sous-vide meat cooked at 60 °C compared with 70 °C. However, as shown in the present study, this did not affect overall acceptability, which was higher for sous-vide beef.

**Table 3:** Sensory quality of *longissimus lumborum* (LL) and *semimembranosus* (SM) steaks aged for 14 days after thermal treatment (scale 1–10 points)

Attribute	Muscle	Thermal treatment	
		Sous-vide	Steam-cooking
Colour—uniformity (surface)	LL	6.70 (0.22)^ax^	6.70 (0.25)^ax^
	SM	7.26 (0.18)^ax^	6.77 (0.22)^ax^
Meat aroma intensity	LL	5.16 (0.26)^by^	6.34 (0.27)^ax^
	SM	6.72 (0.16)^ax^	6.30 (0.21)^ax^
Meat aroma acceptability	LL	5.55 (0.26)^by^	6.60 (0.27)^ax^
	SM	6.88 (0.16)^ax^	6.55 (0.21)^ax^
Juiciness	LL	6.38 (0.24)^ax^	5.24 (0.19)^bx^
	SM	6.46 (0.23)^ax^	4.45 (0.18)^by^
Tenderness	LL	6.70 (0.29)^ax^	5.20 (0.25)^bx^
	SM	7.16 (0.19)^ax^	4.82 (0.16)^bx^
Meat taste intensity	LL	6.42 (0.24)^ax^	6.48 (0.25)^ax^
	SM	7.02 (0.20)^ax^	6.20 (0.20)^bx^
Taste acceptability	LL	6.52 (0.23)^ax^	6.14 (0.20)^ax^
	SM	6.98 (0.16)^ax^	6.07 (0.21)^bx^
Overall acceptability	LL	6.54 (0.23)^ay^	5.42 (0.17)^bx^
	SM	7.12 (0.18)^ax^	5.12 (0.13)^bx^

^a^^−^^b^mean values in rows with different letters differ significantly at *P* < 0.05; ^x.y^mean values in columns within each attribute with different letters differ significantly at *P* < 0.05

**Table 4:** Influence of muscle and thermal treatment method on the quality of 14-d aged beef steaks (mean values and standard error of the mean in parentheses; scale 1–10 points)

Attribute	Muscle (M)		Thermal treatment (T)		*P* level		
	LL	SM	Sous-vide	Steam-cooking	M	T	M x T
Colour—uniformity (surface)	6.70 (0.17)	6.99 (0.15)	6.98 (0.14)	6.74 (0.17)	NS	NS	NS
Meat aroma intensity	5.75 (0.18)	6.49 (0.14)	5.94 (0.17)	6.32 (0.16)	***	*	**
Meat aroma acceptability	6.07 (0.19)	6.70 (0.13)	6.21 (0.17)	6.57 (0.17)	***	**	***
Juiciness	5.81 (0.16)	5.36 (0.17)	6.42 (0.16)	4.81 (0.14)	*	***	NS
Tenderness	5.95 (0.21)	5.88 (0.17)	6.93 (0.18)	4.99 (0.14)	NS	***	NS
Meat taste intensity	6.45 (0.17)	6.57 (0.15)	6.72 (0.16)	6.33 (0.16)	NS	*	*
Taste acceptability	6.33 (0.15)	6.48 (0.14)	6.75 (0.14)	6.10 (0.15)	NS	***	NS
Overall acceptability	5.98 (0.15)	6.03 (0.14)	6.83 (0.15)	5.25 (0.11)	NS	***	NS

LL *longissimus lumborum*; SM *semimembranosus*

***P < 0.001; **P < 0.01; *P < 0.05; NS no significant difference

## Conclusion

Results of the present study indicated that all three studied factors, muscle type, ageing, and thermal treatment method, affected WBSF, cooking loss, and colour of beef. However, similarities between LL and SM samples subjected to the same thermal treatment after the same aging time were also noted, which indicated that the quality of both muscles might be similar when at least 9-d ageing is used before sous-vide or steam-cooking. It might be concluded that the most important factor affecting beef quality is thermal treatment method. Sous-vide method results in cooked meat with desirable sensory characteristics, better tenderness, and lower cooking losses than steam-cooking. The problem of lower aroma intensity and acceptability of sous-vide beef might be overcome by adding spices to the meat or sear the steaks before sous-vide. Although ageing beneficially affected WBSF values, the effect was noted only in the case of SM muscle, which suggests that ageing of LL muscle could be finished after 9 days, without any adverse effect on sous-vide and steam-cooked beef quality.
